# Behavioral Modeling in Weight Management: A Global Bibliometric and Content Analysis of Health Belief Model Applications

**DOI:** 10.3390/bs16060892

**Published:** 2026-06-01

**Authors:** Ionela-Andreea Puiu, Brîndușa Lungu, Izabela-Flavia Hejja

**Affiliations:** 1 Department of Applied Economics and Quantitative Analysis, University of Bucharest, 030018 Bucharest, Romania; ionela-andreea.puiu@faa.unibuc.ro; 2 Doctoral School of Economic and Administrative Sciences, University of Bucharest, 030018 Bucharest, Romania; brindusa.lungu@s.unibuc.ro

**Keywords:** Health Belief Model, weight management, bibliometric analysis, content analysis, self-efficacy, cues to action

## Abstract

The Health Belief Model (HBM) is a foundational framework in weight management research, but its predictive power is often limited by inconsistent measurement of its core constructs. This study systematically investigates how methodological practices and contextual factors shape the explanatory power of HBM in weight management research using an integrated bibliometric and content analysis of 19 articles retrieved from the Web of Science database. In doing so, the study makes three contributions: (i) it maps the intellectual structure and thematic evolution of HBM-based weight management research between 2014 and 2025; (ii) it evaluates how core HBM constructs are operationalized across empirical studies; and (iii) it examines how contextual and institutional factors influence the model’s predictive capacity. Bibliometric mapping indicates that the field shows conceptual maturity but remains geographically fragmented, with research concentrated in countries such as China, Iran, and Ethiopia, and limited cross-cultural collaboration. Content analysis reveals an imbalance toward behavioral intention rather than observed behavioral change. Among HBM constructs, self-efficacy emerges as a consistent and modifiable determinant supporting sustained behavior, while cues to action are inconsistently operationalized, limiting their effectiveness. Overall, the reliance on intention-based metrics and regionally bounded research traditions constrains the predictive power of HBM applications in weight management, highlighting the need for context-sensitive, self-efficacy–focused, and autonomy-preserving interventions.

## 1. Introduction

Effective weight management extends beyond clinical intervention and requires a nuanced understanding of the behavioral determinants that drive individual and collective health decisions (Al-Mahzoum et al., 2025; Bray et al., 2017). Although being overweight is often framed as a medical condition, it is increasingly recognized as a systemic behavioral challenge. This perspective has wide-ranging implications for public health systems and economic productivity (CDC, 2023; OECD, 2019). Given the complexity of health-related behavior, structured theoretical models are needed to explain how individuals form and act upon health-related beliefs. Understanding the determinants of such behavior therefore requires frameworks capable of capturing the cognitive and perceptual processes that underlie health-related decision-making.

Within this context, the Health Belief Model (HBM) has emerged as one of the most widely applied theoretical frameworks for explaining health-related behavior, particularly dietary adherence and physical activity (Daddario, 2007; Lungu et al., 2024). By linking beliefs, intentions, and actions, the HBM offers a structured lens for understanding how cognitive evaluations and social signals influence lifestyle decisions (Champion & Skinner, 2008).

Although it was originally developed to explain participation in preventive health programs, its continued application in contemporary research reflects an important functional shift over time (Alamer, 2024). Rather than serving exclusively as a comprehensive theory of behavior change, HBM is more appropriately understood as a conceptual mapping and early-stage diagnostic framework. In this capacity, it facilitates the systematic identification of perception-based vulnerabilities within target populations.

HBM posits that health-related behaviors are influenced by an individual’s perception of susceptibility and severity, as well as by their evaluations of perceived benefits and barriers. Such beliefs are further reinforced by self-efficacy—the confidence in one’s ability to perform a behavior—and cues to action, which function as triggers that activate health behavioral change (Rosenstock, 1974a, 1974b). Despite its conceptual maturity and extensive use in weight management research (Abd El Samad et al., 2025; Al-Haroni et al., 2024; Beressa et al., 2025; Daddario, 2007), the HBM continues to face challenges regarding predictive consistency and the heterogeneity in how its constructs are operationalized across studies (Alamer, 2024; Harrison et al., 1992).

Empirical findings show substantial variability in the explanatory power of HBM constructs across different settings (Carpenter, 2010). This inconsistency often stems from fragmented measurement approaches and a lack of standardized operational definitions, particularly regarding cues to action. As a result, cumulative knowledge building remains limited, and cross-study comparability is weakened (Janz & Becker, 1984). Moreover, the model’s performance appears highly sensitive to contextual and institutional environments, suggesting that cognitive evaluations do not operate independently of broader social structures.

These limitations do not diminish the model’s relevance; rather, they underscore a critical gap in its current application. Persistent variability in findings highlights the need for a more coherent and context-aware use of HBM constructs, particularly in complex behavioral domains such as weight management.

In this regard, the strength of HBM does not necessarily lie in providing a complete explanatory or interventional framework, but in its ability to identify early-stage motivational barriers, perceptual distortions, and adherence risks prior to the implementation of interventions. This “early warning” function is particularly valuable in designing and tailoring health interventions, where anticipating potential points of failure is as important as explaining behavior.

Some scholars have called for greater standardization in the operationalization of HBM constructs; however, such efforts should be approached with caution. Although conceptual clarity is necessary, rigid standardization may obscure meaningful contextual variation, particularly in the study of weight-related behaviors, which are inherently heterogeneous across populations and socio-cultural environments. Accordingly, this study advocates a context-sensitive operationalization of HBM constructs, emphasizing conceptual clarity and adaptability rather than uniform measurement.

Importantly, such contextual sensitivity extends beyond methodological considerations to include institutional and relational factors that shape how health beliefs are interpreted and enacted in practice. In this study, trust in health authorities is not conceptualized as a formal construct of HBM and is not included in the content analysis. Instead, it is introduced as a contextual moderating factor derived from social capital theory (Fukuyama, 1996; Kawachi & Kennedy, 1999), serving as an interpretative lens to explain how HBM constructs—particularly perceived benefits and cues to action—are received and acted upon across different environments.

From a theoretical perspective, trust shapes the conditions under which individuals interpret and evaluate HBM constructs. The rational-choice mechanisms embedded within HBM implicitly assume a baseline level of institutional credibility. In low-trust environments, perceived benefits may be discounted and cues to action provided by health authorities may lose effectiveness, potentially attenuating the model’s explanatory consistency (Kawachi & Kennedy, 1999).

Despite extensive application of HBM in weight management research no comprehensive synthesis has systematically examined how methodological practices and contextual conditions shape the model’s explanatory performance over time, to the best of our knowledge. Existing studies tend to focus on isolated empirical applications that do not assess the evolution of construct operationalization, collaborative networks, and thematic priorities within the field. Consequently, the field lacks a longitudinal integrated assessment that could clarify whether inconsistencies in predictive performance stem from theoretical limitations, methodological fragmentation, or contextual variability.

To address this gap, the present study systematically investigates how methodological practices and contextual conditions influence the explanatory power of HBM in weight management research from 2014 to 2025. Using a combined bibliometric and content analysis approach, the study examines the field’s intellectual structure, the operationalization of HBM constructs, and the contextual determinants of predictive consistency. Building on this methodological framework, the study is guided by three central research questions:

RQ1: How has the intellectual structure, thematic evolution, and collaboration network of HBM-based weight management research developed over the last decade?

RQ2: How are HBM constructs operationalized across empirical studies, and what are the main sources of methodological inconsistency?

RQ3: How do contextual and institutional factors shape HBM’s predictive capacity and practical applicability?

This study makes three contributions to HBM and weight management literature. First, it provides a longitudinal bibliometric mapping of HBM-based weight management research published between 2014 and 2025, identifying the field’s intellectual structure and thematic evolution. Second, it analyzes how HBM constructs are operationalized across empirical studies and highlights key sources of methodological inconsistency. Third, it examines how contextual and institutional factors influence the predictive capacity and practical applicability of HBM in weight management interventions.

## 2. Materials and Methods

### 2.1. Data

To achieve the proposed objectives, a bibliometric dataset was compiled using data retrieved from two major scientific databases, Clarivate Analytics Web of Science (WoS) and Elsevier’s Scopus. These databases provide extensive coverage of peer-reviewed literature across diverse academic fields, enabling a comprehensive and multidisciplinary evaluation of research output on HBM applications for weight management (Falagas et al., 2008; Mongeon & Paul-Hus, 2016). Their rigorous indexing and journal selection criteria ensure the inclusion of high-quality publications, enhancing the reliability of bibliometric analyses (Gavel & Iselid, 2008).

The structured search conducted across the two databases using the query terms “Health Belief Model” OR HBM AND “weight management” initially yielded a total of 506 results. To refine the pool of potentially relevant studies and focus on studies with detailed empirical analyses, successive search phases targeted using structural equation modeling (SEM), specific HBM constructs, and weight-related interventions and outcomes, resulting in 166 articles for further screening.

**Figure 1:**
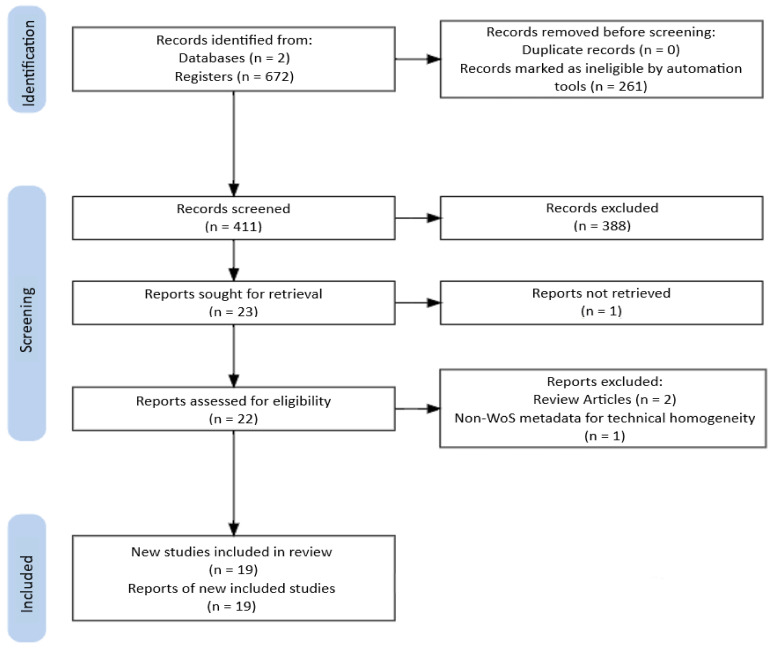
Articles Selection Protocol.

Across all search phases, a total of 672 articles were identified in August 2025. Following the protocol outlined in Figure 1, and applying predefined inclusion and exclusion criteria, the dataset was systematically reduced to a final selection of 20 articles, of which 19 were indexed in WoS and one in Scopus.

To enable bibliometric mapping and network analysis, final refinement was performed to ensure metadata consistency. Bibliometric techniques rely on standardized metadata structures, and combining datasets from different indexing platforms can introduce discrepancies in citation formats, author affiliations, and keyword indexing, potentially affecting the reliability of network construction and comparative analysis (Mongeon & Paul-Hus, 2016). Given that 95% of the relevant articles were from WoS, the bibliometric analysis was restricted to WoS-indexed studies to maintain dataset homogeneity and methodological consistency. The inclusion of a single Scopus-indexed article would not have enabled a meaningful cross-database comparison, and this limitation is explicitly acknowledged when interpreting the results.

The use of SEM-focused search terms was intended to identify studies employing robust empirical analyses of HBM constructs, while acknowledging that this approach may have excluded some descriptive or qualitative HBM studies.

Furthermore, to ensure rigor in content analysis, a standardized codebook was developed a priori. Each study was independently coded by two reviewers, with inter-rater reliability assessed using Cohen’s kappa (see Appendix A). Overall agreement was substantial (κ = 0.753, Po = 0.835), with construct-level kappa values ranging from 0.712 to 0.782, all within the substantial agreement range (Landis & Koch, 1977). Any discrepancies between coders were resolved through discussion and consensus. Variables were classified on an ordinal scale from 0 to 3 to quantify operationalization and statistical significance, with detailed systematic coding presented in Appendix B.

### 2.2. Methods

The research methodology adopts a complementary approach combining bibliometric and content analysis.

Bibliometric analysis provides a systematic evaluation of global research trends, publication dynamics, and the most influential contributors within the domain of HBM and weight management (Aria & Cuccurullo, 2017; Donthu et al., 2021). It focuses on two main approaches: performance analysis and science mapping (Donthu et al., 2021). Performance analysis assesses research impact and productivity through article-related metrics, the scientific output by countries and regions, the productivity of authors and institutions, as well as publishing sources and keyword trends. Science mapping identifies thematic clusters and intellectual structures within the field by performing co-authorship, co-citation and co-word analyses (Puiu & Bîlbîie, 2025). These analyses were performed using the bibliometrix package (Aria & Cuccurullo, 2017) and Biblioshiny web application (R Core Team, 2023) within the R software environment (version 4.4.1 for Windows).

In addition to the bibliometric approach, content analysis was conducted to obtain a more in-depth understanding of how HBM has been implemented and operationalized in the relevant literature. Specifically, this analysis aimed to identify the major themes related to HBM and weight management and to develop categorical frameworks describing both the content and methodological approaches used in HBM-based research and weight management interventions. This approach provides further insight into how HBM has been applied in practice from its theoretical foundations.

## 3. Bibliometric Analysis

### 3.1. Performance Analysis

Performance analysis in bibliometric research relies on quantitative indicators to evaluate scholarly contributions across multiple dimensions, including authorship, journals, institutional affiliations, publication outputs, as well as citation-based impact metrics (Donthu et al., 2021; Cucari et al., 2023).

#### 3.1.1. Publications Related Metrics

It was found that the WoS dataset spans publications from 2014 to 2025 and covers a wide range of journals. BMC Public Health emerged as the most prominent publishing source, contributing two articles, with the remaining 17 journals publishing one study each. Although this distribution indicates a variety of publication venues, all identified journals fall within the health sciences, suggesting that the publication landscape reflects diversity across health-related subfields rather than genuine interdisciplinary engagement beyond the health domain.

The documents have an average age of 3.68 years, ranging from 0 to 11 years, indicating a strong emphasis on recent publications. The average number of citations per document is 17.11. The most influential study examined the adoption of fitness mobile applications (Wei et al., 2021), published in the journal Health Communication, and has received 94 citations, averaging 18.8 citations per year.

Research output comprises 81 authors, with an average of 4.26 co-authors per paper. Notably, no single-authored publications were identified. Despite this level of collaboration, the international co-authorship rate remains low (15.79%), indicating that research activities remain largely localized. This pattern suggests that, although studies benefit from a strong internal collaboration, global intellectual networks remain fragmented–a phenomenon commonly observed in complex public health challenges such as weight management, where HBM is often applied in local rather than a global context.

Table 1 presents the descriptive statistics of the articles included in the database.

**Figure 2:**
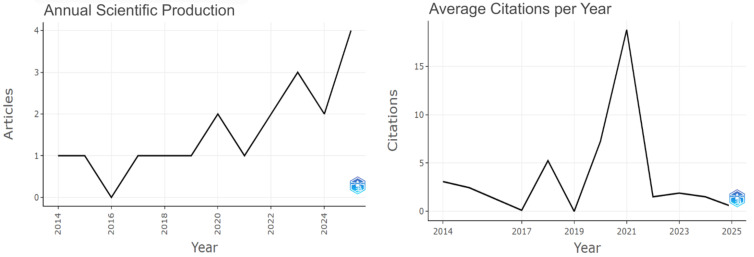
Research progress between 2014 and 2025.

The data reveal a fluctuating yet overall upward trend in annual publication output. After a period of sporadic output from 2014 to 2019, research productivity increased substantially in 2020. Output peaked in 2025 with four publications, preceded by three articles in 2023. Figure 2 illustrates the temporal evolution of the publication output.

Additionally, analysis of average citations per year reveals a distinct pattern (Figure 2). The most influential articles were published in 2021 (18.80 average citations) and 2020 (7.25 average citations). This pattern is consistent with established academic trends, where more recent publications have had less time to accumulate citations than older, foundational works.

#### 3.1.2. Citation Analysis

Citation analysis illustrates intellectual interconnections and influence in a research field (Hulland, 2024; Passas, 2024). Within this dataset, PLOS One is the most frequently cited journal (28 citations), followed by the International Journal of Environmental Research and Public Health (19 citations). Other prominent sources include BMC Public Health, Health Education Quarterly, and the Journal of Nutrition Education and Behavior, each with 13 citations. Local citation analysis further identifies the work of Saghafi-Asl et al. (2020) and McArthur et al. (2018) as the most influential references within the dataset. These highly cited studies primarily examine the application of HBM constructs, particularly self-efficacy and perceived barriers, in digital and community-based weight loss interventions.

#### 3.1.3. Collaboration Analysis

**Figure 3:**
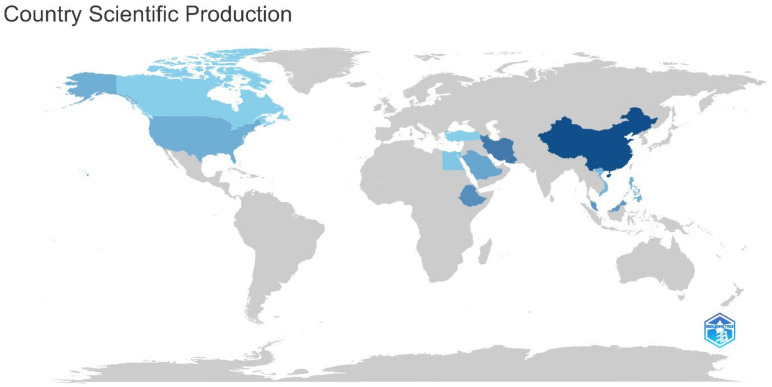
Scientific Production by Country.

Collaboration analysis illustrates the structural and cooperative dynamics within a research community (Passas, 2024). As shown in Figure 3, research on HBM and weight management show clear geographical concentration. China leads scientific output with 18 publications, followed by Iran and Ethiopia. Notable contributions also emerge from Malaysia, Saudi Arabia, the Philippines, and the United States, indicating that HBM constructs are being applied across diverse cultural and geographic contexts. Figure 3 presents the distribution of scientific production by country.

A different hierarchy emerges when total and mean citations per article are considered (Figure 3). China records the highest total citation count of 146, whereas the United States demonstrates the highest average citations per article, at 39.50. This high citation density likely reflects the central role of U.S. institutions in global scientific networks, as well as the broad visibility afforded by large-scale funding supporting research on foundational public health issues (Aria & Cuccurullo, 2017). Similarly, Iran’s average of 29.30 citations, slightly higher than China’s 29.20, suggests a degree of regional influence. This pattern may be attributed to a recent growth in Iranian medical research productivity and a strategic emphasis on specialized clinical interventions that address significant national health needs, leading to high recognition within regional academic communities (Khanali et al., 2023; Sadeghi-Bazargani et al., 2019).

**Figure 4:**
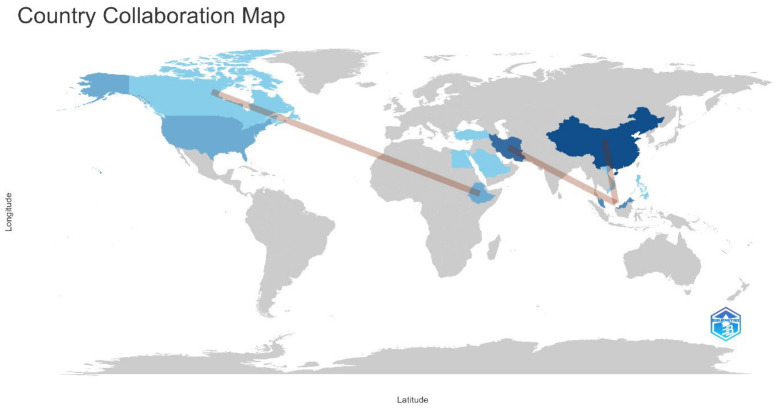
Collaboration World Map by Country.

Cross-country collaboration reveals the primary connections established between China and Malaysia, Ethiopia and Canada, Iran and Malaysia (Figure 4). The limited number of international research partnerships suggests that most studies are conducted within national borders. This pattern may be driven by the strong cultural influences on weight management and the subjective perceptions associated with the HBM (Carpenter, 2010). The prevalence of within-country research limits cross-context comparability, as HBM constructs, such as perceived barriers, are frequently shaped by local socio-cultural and healthcare infrastructures rather than universal behavioral mechanisms (Jones et al., 2015). Although this localization enhances intervention specificity, it simultaneously fragments global intellectual networks. Integrating these collaboration patterns with epidemiological data on weight problem trends in countries such as China and Iran may help determine whether these localized collaborations represent strategic institutional responses to shared regional health challenges (Abarca-Gómez et al., 2017). The global distribution of cross-country collaborations is illustrated in Figure 4.

### 3.2. Science Mapping

Science mapping employs mathematical methods to visualize and analyze the structural evolution of a scientific domain (Chen et al., 2014). This approach integrates co-citation, co-word, and co-authorship analyses (Donthu et al., 2021) to assess research productivity and the collaborative connectivity of authors, institutions, and countries (Durieux & Gevenois, 2010; Vâlsan et al., 2024).

#### 3.2.1. Social Structure—Co-Authorship Analysis

Co-authorship analysis maps the interaction patterns and collaborative structures within a research community (Donthu et al., 2021). By identifying researchers who share scientific backgrounds and geographical affiliations, this method reveals the social organization of the field (Passas, 2024), although these insights strongly depend on network density and dataset size.

**Figure 5:**
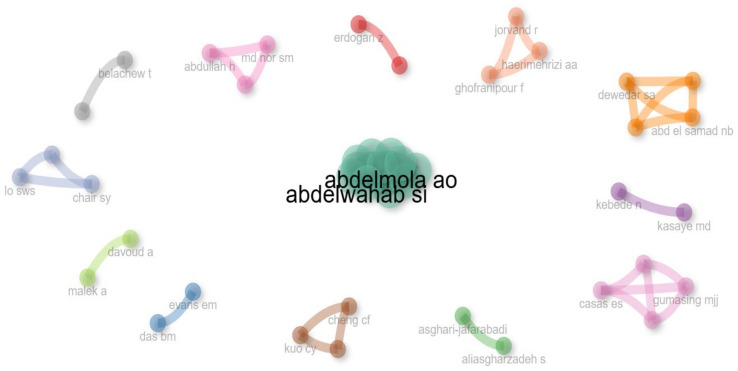
Co-authorship clusters.

Our analysis identified 13 distinct clusters, each reflecting varying degrees of geographical and disciplinary specialization. Given the dataset size (19 articles), the resulting network exhibits low density with a limited number of co-authorship ties. In such low-density networks, structural segmentation is expected, as even minor differences in authorship relations can influence the network configuration and result in variations in network structure. The clusters are presented in Table 2 and illustrated in Figure 5.

The first cluster (red) is geographically limited to Turkey and comprises researchers specializing in general and internal medicine. The second cluster (blue) represents an interdisciplinary research group from the United States, focusing on sport sciences and psychology. The third cluster (green), limited to Iran, includes experts in nutrition, dietetics and public health. The fourth cluster (purple) consists of Ethiopian collaborators working across healthcare sciences, public health, and technology.

The fifth cluster (orange), confined to Egypt, focuses on environmental health and ecology. The sixth cluster (brown) is localized in Taiwan, with researchers specializing in biochemistry, pharmacology, and nutrition. The seventh cluster (pink) includes Malaysian scholars integrating business, social sciences, and public health. The eighth cluster (grey) represents a second Ethiopian group with a focus on science, technology, and nutrition.

The ninth cluster (mint green) consists of Saudi Arabian-based scholars with interests in internal medicine, biomedical social sciences, and psychiatry. The tenth cluster (dark orange) is a second Iran-based cluster focused on public health and nursing. The eleventh cluster (dark blue) represents a collaborative team from China specializing in nursing and cardiology. The twelfth cluster (dark pink) includes scholars from the Philippines scholars bridging food science and computer science. The thirteenth cluster (lime green) represents a third Iran-based cluster focused on health care sciences and services.

The predominance of geographically bounded clusters suggests that collaboration patterns are primarily localized rather than global. Instead of indicating distinct or stable research communities, the observed structure reflects limited cross-national collaboration within the dataset. This aligns with the overall relatively low international co-authorship rate (15.79%), which indicates that most collaborative activity occurs within national rather than international contexts.

Overall, the co-authorship structure should be interpreted as an exploratory representation of collaboration patterns in a limited dataset rather than a definitive mapping of the field. Analyses based on larger and more heterogeneous datasets are needed to determine whether the observed structural fragmentation persists or whether more integrated collaboration networks emerge under broader sampling conditions.

This pattern reflects a multidisciplinary orientation in HBM-related weight management research, emphasizing applied and practice-oriented approaches without implying a directional shift in the field.

#### 3.2.2. References Co-Citation Analysis

Co-citation analysis reveals the intellectual structure of a research field by grouping publications that are frequently cited together (Donthu et al., 2021), thereby identifying foundational themes and seminal works (Hulland, 2024; Passas, 2024). This method maps how knowledge is constructed around shared ideas by identifying thematic clusters composed of interconnected nodes and edges (Fusco et al., 2020).

**Figure 6:**
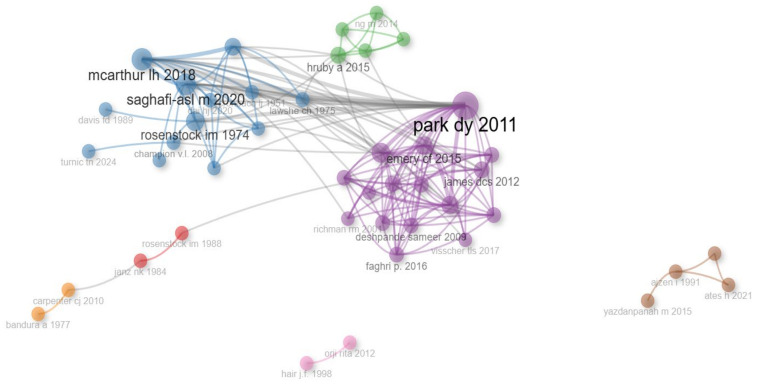
Co-citation analysis of references.

The co-citation analysis was conducted using 50 nodes and the Walktrap clustering algorithm (Puiu & Bîlbîie, 2025; Lancichinetti & Fortunato, 2009). The resulting network revealed three central nodes: “park dy 2011” (Park, 2011) (betweenness centrality = 295.633), “mcarthur lh 2018” (McArthur et al., 2018) (betweenness centrality = 166.405) and “saghafi-asl m 2020” (Saghafi-Asl et al., 2020) (betweenness centrality = 148.595), as illustrated in Figure 6. Park (2011) applied the HBM to predict female middle school students’ intentions to reduce weight, considering their weight status. McArthur et al. (2018) examined college students’ weight-related beliefs to evaluate the predictive power of the HBM for body mass index (BMI). Finally, Saghafi-Asl et al. (2020) explored factors influencing behavioral intentions related to general weight management.

The co-citation analysis yielded seven clusters, as shown in Figure 6. The largest cluster, Cluster 4 (purple), includes 16 papers that apply HBM and its core constructs, self-efficacy, perceived benefits and perceived barriers, to predict weight-related behaviors and health outcomes across diverse populations. The second largest cluster, Cluster 2 (blue), comprises 13 papers focusing on HBM-based health interventions design and social determinants of weight management. This cluster bridges theory and practice by addressing global obesity risks and highlighting efforts to translate theoretical insights into targeted public health actions.

The third cluster, Cluster 3 (green), consists of five papers examining the global epidemiological and economic dimensions of obesity. This group combines analyses of worldwide obesity prevalence trends with research on the economic associated with the condition and studies on specific vulnerable groups, such as college freshmen. The fourth cluster, Cluster 6 (brown), includes four papers focused on integrating HBM with the Theory of Planned Behavior (TPB). This cluster represents an advanced theoretical approach, using TPB as a framework to enhance the predictive power of the HBM in examining specific health behaviors, including healthy eating and cancer screening.

The remaining clusters comprise two papers each, highlighting the theoretical and methodological nuances of the field. Cluster 1 (red) traces the early conceptual evolution of HBM, linking it to the Social-Learning Theory through a retrospective analysis that underscores the field’s intellectual roots. Cluster 5 (orange) focuses on integrating self-efficacy into predictive health behavior models. Finally, Cluster 7 (pink), examines methodological extensions of the HBM, using multivariate data analysis to enhance the model’s practical application in health intervention design. The co-citation network reveals a mature intellectual structure centered on three foundational studies (McArthur et al., 2018; Park, 2011; Saghafi-Asl et al., 2020) that bridge theoretical evolution with practical weight management. These studies are organized into seven thematic clusters, transitioning from core HBM constructs and social determinants to integrations with the TPB.

#### 3.2.3. Conceptual Structure—Co-Word Analysis

Co-word analysis is a technique that examines the co-occurrences of core concepts within the keywords, abstracts, or titles of publications (Vâlsan et al., 2024), based on the premise that concepts that frequently co-occur are thematically related (Passas, 2024). This method allows for the visualization of conceptual clusters within a research field (Donthu et al., 2021).

To visualize these clusters, we used a thematic map, plotting the bigram terms from the paper abstracts into four quadrants based on centrality and density scores (Vâlsan et al., 2024; Cobo et al., 2011, 2015). Centrality indicates the level of interaction between networks and quantifies the importance of a concept within a research field, while density reflects a group’s internal strength and indicates the level of development of a specific topic (Cobo et al., 2011, 2015; Puiu & Bîlbîie, 2025). The size of each cluster corresponds to the terms’ frequency and the volume of connected documents within the group (Fusco et al., 2020).

**Figure 7:**
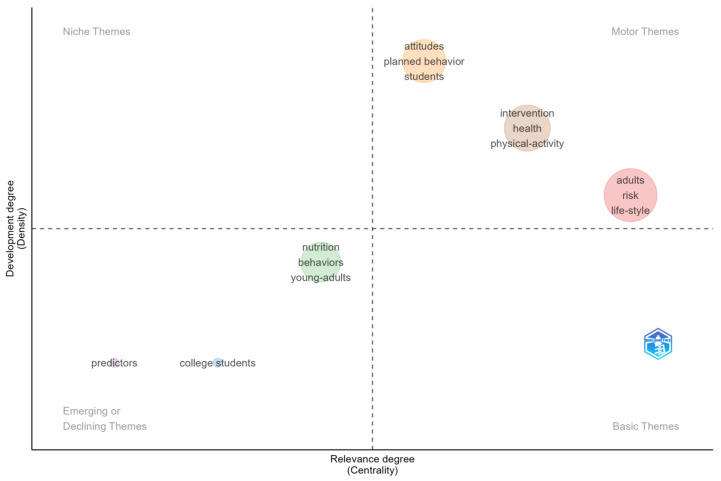
Co-word analysis.

The thematic map organizes conceptual clusters into four strategic groups: motor, niche, emerging/declining, and basic themes (Cobo et al., 2015). To reduce self-referential bias, query terms and their synonyms were excluded, allowing the thematic map to reveal the field’s underlying conceptual structure. The results of the co-word analysis are illustrated in Figure 7.

The first cluster represents motor themes, corresponding to a well-developed and highly central research area (Cobo et al., 2011). This quadrant contains three motor themes. The first, centered on “adults” (centrality = 1.278; density = 73.889), reflects research applying the HBM to address health risks and lifestyle modifications in adult populations. The second, focused on “intervention” (centrality = 1.111; density = 77.083), highlights the model’s practical utility in designing physical activity-centered health improvements. The third motor theme “attitudes” (centrality = 0.750; density = 93.750), emphasizes the integration of the HBM with other psychological frameworks, such as TPB, particularly in studies involving younger academic populations.

The third quadrant represents emerging or declining themes, characterized by low centrality and density (Cobo et al., 2011). The presence of “predictors” (centrality = 0.250; density = 50.000) indicates an emerging research stream focused on identifying variables beyond the HBM to enhance the model’s predictive utility. Furthermore, the positioning of “nutrition” (centrality = 0.694; density = 63.889) within this quadrant suggests that the research of nutritional patterns is an evolving theme that is gradually gaining structure but not yet fully integrated into the field’s core conceptual network.

The second and fourth quadrants, representing niche (internally well-developed but specialized) and basic (fundamental and broadly connected to other topics) themes, respectively (Cobo et al., 2011), are empty. The absence of niche themes suggests a high level of disciplinary fluidity, in which specialized knowledge is readily integrated into the mainstream rather than remaining isolated. Similarly, the lack of distinct basic themes indicates theoretical consolidation, as foundational concepts have become so deeply embedded within motor themes that they no longer exist as separate, diffused elements. Together, these patterns reflect a mature research landscape, characterized by a smooth transition from fundamental theory to complex, well-integrated applications.

The conceptual structure indicates thematic maturity, with specialized knowledge and foundational theories integrated into the core themes. However, this bibliometric perspective may mask a lack of true interdisciplinary integration. While the co-occurrence of terms, such as “physical activity”, “lifestyle”, and “planned behavior” suggests a broad application of the HBM, it does not clarify how these constructs are operationalized or whether their integration leads to measurable behavioral changes.

The strategic map identifies the “where” and “what” of the research but provides limited insight into how the model is applied and implemented. The absence of niche themes may indicate either a theoretically mature field or a limited degree of specialized innovation, as well as a reliance on generic applications of the model. To determine whether these integrated themes reflect a genuinely multidisciplinary framework or merely a cluster of related yet isolated studies, we need to look beyond term frequency. Accordingly, a structured content analysis is employed to systematically examine the methodological rigor, depth of theoretical integration, and practical effectiveness of the reported interventions. The findings of this qualitative examination are detailed in the following sections.

## 4. Content Analysis

Building on the structural patterns identified through bibliometric mapping, this content analysis shifts the focus from a macro-level overview of the research landscape to a micro-level examination of how HBM is operationalized in practice. Rather than mapping the architecture of the field, the analysis critically evaluates methodological rigor, depth of theoretical integration, and practical applicability of the selected studies within their operationalization. Importantly, the content analysis focuses exclusively on explicitly reported HBM constructs and their operationalization. Contextual factors such as trust are not coded as variables but are discussed interpretively when they emerge as explanatory mechanisms within literature.

This qualitative appraisal focuses on three critical dimensions: construct integration, behavioral relevance, and intervention efficacy. By examining the mechanics underlying intention-based modeling and adaptive behavior, the analysis highlights the field’s epistemic strengths and methodological vulnerabilities. In doing so, it evaluates the HBM’s capacity to inform real-world interventions and support sustained behavioral adherence, thereby bridging the gap between theoretical constructs and practical effectiveness.

To enhance transparency and methodological rigor, a structured synthesis table was developed to systematically map the operationalization of HBM constructs across the included studies. The table synthesizes information on measurement instruments, reliability, statistical methods, and behavioral outcomes, allowing for a direct comparison of methodological approaches and construct-level heterogeneity across the dataset.

The synthesis presented in Appendix C reveals several recurring methodological patterns across the included studies derived from coded empirical variables (constructs, measurement, reliability, statistical methods, and outcomes). Although the core constructs of HBM are consistently represented, their operationalization differs markedly with respect to measurement instruments, scale composition, and the degree of validation. While some studies rely on established or theory-driven questionnaires, others employ adapted or study-specific tools, often with limited reporting of psychometric properties.

Reporting of reliability is similarly uneven. Cronbach’s alpha values, where provided, generally fall within moderate to high ranges; however, several studies do not report reliability at all. This inconsistency complicates the assessment of internal validity and limits comparability across studies.

Methodological diversity is also evident in the statistical techniques applied, ranging from basic correlational and regression analyses to more advanced SEM approaches. Despite these differences, most studies share common design features, notably cross-sectional frameworks and reliance on self-reported data, both of which introduce potential bias and limit the ability to infer causal interpretation.

Taken together, these observations point to a heterogeneous operationalization of HBM constructs. Conceptually similar components are measured using diverse and not always equivalent approaches, which may contribute to variability in reported findings. Greater consistency in measurement and reporting practices would strengthen the comparability and cumulative value of future research in this area.

Against this methodological backdrop, an additional pattern emerges regarding outcome specification.

### 4.1. Intention-Centered Modeling

This section analyzes outcome specification and modeling strategies as coded dimensions of the included studies, focusing on the relationship between HBM constructs and reported behavioral outcomes.

The systematic content analysis of the selected studies reveals that, although the core HBM constructs, perceived susceptibility (n = 5), perceived severity (n = 6), perceived benefits (n = 7), and perceived barriers (n = 8) are commonly referenced across studies, their operationalization remains fragmented and methodologically inconsistent. In addition to this variability in measurement, a broader structural pattern emerges, whereby most studies prioritize behavioral intention as the primary outcome variable.

Six studies are exceptions (Das & Evans, 2014; Jorvand et al., 2020; Lo et al., 2015; McArthur et al., 2018; Nategh et al., 2017; Sheng et al., 2023), while the remaining studies rely on self-reported intention as a proxy for actual behavior. Although this emphasis aligns with the cognitive foundations of HBM, intention is rarely supported by empirical evidence of sustained behavioral adherence.

Within this intention-centered framework, SEM emerges as the dominant analytical technique. Eight studies employed SEM to estimate relationships among HBM constructs and weight management intentions (Beressa et al., 2025; Espeño et al., 2024; Jorvand et al., 2020; Md Nor et al., 2025; Saghafi-Asl et al., 2020; Sheng et al., 2023; Y. Wang et al., 2022; Wei et al., 2021). However, comparative methodological analysis shows substantial variation in the depth of SEM application, ranging from basic path analysis to more advanced latent variable modeling with formal model-fit assessment (Y. Wang et al., 2022).

This methodological heterogeneity limits cross-study comparability, making it difficult to draw consistent conclusions regarding the relationship between intention and behavior.

However, intention-based modeling may not sufficiently capture the complexities of sustained behavioral adherence, particularly under conditions shaped by habit, environmental constraints, and psychosocial stressors. Reliance on intention as a proxy outcome reduces the applicability of these findings for the design of effective long-term interventions (Borges-Tiago et al., 2024; Conner & Norman, 2022; Enste & Altenhöner, 2021).

### 4.2. Self-Efficacy as a Core Determinant for the Adaptive Capacity in Behavioral Models

Beyond the limitations of intention-centered modeling, the content analysis identifies self-efficacy as a central determinant of adaptive behavioral capacity. Across diverse demographic, technological, and cultural contexts, self-efficacy emerges not merely as an auxiliary factor (Raude et al., 2020), but as a core driver of individuals’ ability to translate intentions into sustained action (Bandura, 1982, 1986; Code, 2020; Maddux, 2013).

Self-efficacy exhibits strong predictive power across a variety of contexts, ranging from digitally enhanced interventions (Jorvand et al., 2020) to gender-sensitive dietary models (Ahmad et al., 2023) and culturally adapted HBM frameworks (Vo & Le, 2025). The reviewed studies further identify self-efficacy as a modifiable and mediating construct that enables individuals to translate health beliefs into sustained adherence, when supported by organizational, relational, and contextual resources. This aligns with Social Cognitive Theory (SCT) and subsequent empirical evidence, underscoring self-efficacy’s central role in bridging cognitive intention and real-world action.

The reviewed studies operationalize self-efficacy through a range of methodological approaches, ranging from direct self-assessment (Das & Evans, 2014) to SEM (Wei et al., 2021), reflecting its central role in explaining behavioral adherence. Self-efficacy interacts with other HBM constructs—perceived benefits, perceived barriers, and threat appraisal—to shape both intention formation and the likelihood of real-world behavioral execution.

Within integrated frameworks, such as HBM, TPB, and SCT, self-efficacy functions as a behavioral enabler (Bandura, 1977). It supports the translation of beliefs into action within HBM contexts, (Kebede et al., 2023), converts intention into execution within TPB (Ajzen, 1985; Beressa et al., 2025), and sustains behavior under challenging conditions within SCT (Bandura, 1986). In this sense, self-efficacy operates as an intervening mechanism between intention and action, mediating the relationship between cognitive evaluation and real-world adherence.

Consistent with prior theoretical and empirical work, our analysis indicates that self-efficacy is central to behavioral change and adaptive capacity. Its mediating role underscores the importance of supporting self-efficacy through organizational, relational, and contextual resources, thereby enhancing the translational relevance and long-term effectiveness of HBM-based interventions.

### 4.3. Cues to Action as Behavioral Catalysts: Addressing Fragmentation and Structural Underspecification

The analysis of the 19 studies reveals a heterogeneous and conceptually dispersed approach to the operationalization of cues to action within health behavior models, with 14 of the 19 studies addressing this construct to some degree. Of these, only seven found cues to action statistically significant, while three measured the construct without reaching significance and four mentioned it only qualitatively. Five studies did not include cues to action at all. Specifically, operationalization varied considerably across studies: Saghafi-Asl et al. (2020) and Vo and Le (2025) used structured subscales distinguishing internal cues (e.g., physical symptoms, emotional states) from external ones (e.g., media messages, physician advice); Hou et al. (2022) framed a vegetarian meal plan as an embedded behavioral cue predicting measurable body composition change; Sheng et al. (2023) operationalized peer influence as a social cue mediating physical activity gains; while Nategh et al. (2017) relied on a single-item measure, and Espeño et al. (2024) found that involuntary cues had negligible influence compared to deliberative factors.

Despite its theoretical centrality, cues to action are neither consistently defined nor uniformly measured across empirical contexts. As noted above, several studies employed structured subscales differentiating between internal and external cues (Saghafi-Asl et al., 2020; Vo & Le, 2025), thereby preserving the theoretical distinction embedded in HBM, while others relied on single syntagms (Nategh et al., 2017) or indirect proxies such as social support, media exposure, or institutional messaging, extending the construct beyond its original conceptual perimeter without explicit theoretical justification. This variability reflects more than methodological diversity; it indicates structural fragmentation in which motivational constructs are incorporated into broader behavioral models without consistent conceptual stabilization. As a result, comparability across studies is substantially constrained, and a coherent epistemic consensus regarding the construct’s boundaries remains absent. The theoretical anchoring of cues to action within HBM, TPB, or hybrid frameworks therefore appears partial, inconsistent, and occasionally implicit rather than systematically articulated. Such inconsistency limits cumulative theory building and constrains the capacity to derive integrative conclusions across empirical settings.

When behavioral constructs are operationalized as static inputs rather than emergent properties of complex systems, structural under specification can result. In such contexts, cues to action risk being narrowly interpreted as external triggers, detached from the institutional logic and cultural frameworks that confer meaning. This reductionist approach may generate misaligned strategies, privileging superficial reminders over deeper motivational architectures capable of sustaining long-term lifestyle change.

Although several studies (Abd El Samad et al., 2025; Kebede et al., 2023; Raman et al., 2023; Vo & Le, 2025) adapted measurement instruments from prior literature, concerns regarding ecological validity and cultural sensitivity remain salient. These limitations underscore the need to reconceptualize cues to action as embedded governance signals, whose effectiveness depends on institutional coherence, perceived legitimacy, and alignment with broader resilience strategies (Fukuyama, 1996; Vâlsan et al., 2025).

Moreover, evidence from other studies (Das & Evans, 2014; Sheng et al., 2023) indicates that cues to action frequently operate through emotionally resonant, socially embedded mechanisms, including peer influence, financial incentives, and digital nudges. This suggests that activation processes are mediated not only cognitively but also affectively and relationally. Such findings reinforce the view that cues to action function as behavioral interfaces shaped by affective heuristics, collective norms, and structural design.

From an interpretive perspective, these findings suggest that cues to action may function as dynamic mediators within complex behavioral ecosystems. Refining their conceptualization in this direction would enhance theoretical clarity, strengthen methodological coherence, and support the development of interventions that move beyond isolated, short-term activation cues toward structurally sustained behavioral change.

### 4.4. Methodological Convenience and Its Epistemic Costs

The methodological landscape across the analyzed studies reveals a range of statistical techniques, reflecting both the complexity of health behavior modeling and the fragmented operationalization of cues to action.

Eight studies employed SEM to explore latent constructs, path coefficients, and model fit indices (Beressa et al., 2025; Espeño et al., 2024; Jorvand et al., 2020; Md Nor et al., 2025; Saghafi-Asl et al., 2020; Sheng et al., 2023; Y. Wang et al., 2022; Wei et al., 2021). In these cases, SEM provided a framework for modeling latent structures and capturing multidimensional behavioral dynamics.

Other studies relied on multivariate regression techniques, including linear, logistic, and hierarchical models, to evaluate the predictive power of HBM constructs and demographic variables on outcomes such as BMI and physical activity levels (Albasheer et al., 2024; Hou et al., 2022; Kebede et al., 2023; Lo et al., 2015; McArthur et al., 2018; Raman et al., 2023; Saghafi-Asl et al., 2020; Vo & Le, 2025). Several investigations employed non-parametric tests (e.g., Kruskal–Wallis, Mann–Whitney U, and chi-square) or ANOVA to assess group differences and validate model assumptions (Abd El Samad et al., 2025; Albasheer et al., 2024; Jorvand et al., 2020; McArthur et al., 2018; Raman et al., 2023; Saghafi-Asl et al., 2020). Notably, Das and Evans (2014) used qualitative Nominal Group Technique sessions to explore motivational heuristics, while Nategh et al. (2017) and others limited their analyses to Pearson correlation and reliability testing without implementing comprehensive structural models.

This methodological heterogeneity underscores both the strengths and the limitations of current modeling practices. Although SEM provides a robust mechanism for examining latent behavioral dynamics, its inconsistent application and reliance on adapted instruments without contextual recalibration raise concerns regarding construct validity and theoretical coherence. In several cases, SEM appears to function more as a procedural technique than a conceptually grounded modeling strategy (Jorvand et al., 2020; Espeño et al., 2024), resulting in latent constructs that are insufficiently anchored in behavioral reality.

More broadly, the analytical patterns suggest a tendency toward methodological convenience. While pragmatically justified, the frequent reliance on regression-based and nonparametric approaches prioritizes accessibility over structural depth. The relatively limited engagement with path modeling and comprehensive structural frameworks indicates a constrained exploration of the causal architecture underlying health behavior. Consequently, many models are statistically robust yet insufficiently grounded in theory, yielding findings that are methodologically sound but behaviorally limited.

### 4.5. The Long Road from Mapping Predictors to Shaping Public Policy

Within our dataset, only 1 study out of 19 employed a composite framework integrating TPB, HBM, and the Theory of Effort Minimization in Physical Activity (TEMPA), highlighting the limited use of such integrative models (Espeño et al., 2024). The remaining 18 articles relied on single-framework, intention-based models. At the same time, none of the studies incorporated feedback loops, temporal dynamics, or adaptive behavioral processes. Identity-driven influences, cultural factors, socioeconomic constraints and deeper motivational elements were rarely addressed and generally remained under-specified. These patterns suggest that the field’s conceptual narrowness is empirically observable.

This configuration reflects a research tradition built around survey-based models that presume stable preferences and linear causality. The articles also largely overlook scarcity pressures, institutional trust and the adaptive strategies individuals deploy under uncertainty. No study referenced behavioral pressure systems shaping compliance, resistance or disengagement, despite evidence that stress can undermine cognitive decision-making models. Likewise, no study examined how identity may override intention or how low-trust environments may trigger defensive behavior. Importantly, these observations reflect gaps in empirical literature rather than variables derived from the coded content analysis. This suggests that the observed methodological homogeneity may be structurally produced.

TEMPA offers partial leverage through perceived effort and reward salience. However, its application rarely integrates structural dimensions necessary for designing policies resilient to behavioral variability.

Consequently, public-policy implications remain limited because the evidence base is overly uniform and insufficiently contextualized.

## 5. Discussion

The bibliometric and content analysis of the HBM-based studies in weight management (2014–2025) reveals a field at a turning point. While HBM remains a widely used and effective framework for predicting behavioral intentions, our findings suggest that it frequently overlooks structural and environmental factors that shape actual behavior change. These findings are structured according to the study’s three research questions, addressing (i) bibliometric structures, (ii) operationalization of HBM constructs, and (iii) contextual and methodological implications for intervention design.

Our bibliometric mapping of scientific networks highlights patterns in global research clusters. The relatively low level of international collaboration suggests that HBM research remains largely within national borders, likely due to the highly subjective and culturally influenced nature of weight management behaviors (Albasheer et al., 2024). Notably, the most intensive collaborative activity is concentrated in regions experiencing rapid increases in obesity prevalence, such as the United States, parts of East Asia, and the Middle East.

Correlating these clusters with regional health data reveals a pattern. In countries such as China and Iran, which are experiencing rapid increases in obesity and metabolic disorders (Popkin et al., 2020), localized collaborations appear to function as context-specific, policy-oriented research networks aligned with regional health needs. For instance, the China-Malaysia cluster focuses on urban student populations, where rapid lifestyle changes have outpaced public health infrastructure. Unlike studies conducted in Western contexts, these Middle Eastern and Asian clusters frequently involve public health actors who aim to translate HBM constructs into culturally tailored interventions, addressing local perceived barriers that universal models often overlook (Guan et al., 2025).

Our analysis shows that many studies treat intention as a proxy for behavior; however, behavioral economics demonstrates that intentions are fragile—vulnerable to present bias, friction, and emotional states (Borges-Tiago et al., 2024; Conner & Norman, 2022; Enste & Altenhöner, 2021). This fragility, combined with the limitations of correlational designs, constrains their ability to capture causal mechanisms and the dynamic, context-dependent nature of habitual behaviors such as eating and exercise (Marteau et al., 2021; Roberto & Kawachi, 2015; Webb & Sheeran, 2006). For example, Beressa et al. (2025) measured intention with a seven-item TPB Likert composite and subsequently dichotomized the scores, a choice that may enhance analytical clarity but reduces variability and obscures contextual nuance. Likewise, Espeño et al. (2024) show that when intention models are misaligned with lived constraints, agency-building interventions become necessary to complement intention-based models. Taken together, these limitations suggest that researchers should complement intention-based approaches with environmental architectures that make healthy choices intuitive, alongside agency-focused strategies and longitudinal experimental designs capable of capturing sustained behavior change and unintended consequences (Rutter et al., 2017; Roberto & Kawachi, 2015; Marteau et al., 2021; Parkinson et al., 2025; Schmidt, 2024).

Across the 19 reviewed studies, self-efficacy was explicitly measured in 17 and found statistically significant in 13, making it the most consistently supported HBM construct in the corpus. Compared to broader health beliefs, self-efficacy appears to be more sensitive to contextual and behavioral variation (Jorvand et al., 2020). Unlike static constructs, it adapts to situational constraints and can be strengthened through digital and other context-sensitive interventions. (Abd El Samad et al., 2025; Raman et al., 2023). For example, Beressa et al. (2025) identify self-efficacy as a strategic determinant in youth models (Saghafi-Asl et al., 2020) and maternal studies show that nutrition education improves gestational weight management and birth outcomes via enhanced self-efficacy. Y. Wang et al. (2022) and Vo and Le (2025) find that self-efficacy predicts dietary behavior more strongly than intention, supporting culturally adapted HBM formulations.

Social processes matter: Sheng et al. (2023) show peer support raises self-efficacy and thereby increases student physical activity while Lo et al. (2015) report self-efficacy predicts real-world prevention behaviors in metabolic syndrome. Perceived behavioral control expressed through self-efficacy positions the family as a core determinant of societal resilience, with Md Nor et al.’s integrated model outperforming TPB and HBM in explaining lifestyle adherence (Vâlsan et al., 2025; Md Nor et al., 2025). Within university weight-management settings, asymmetric adaptive agency—some students confident, others more responsive to situational cues—calls for targeted confidence-building and autonomy-supporting interventions; absent such agency-focused architectures, readiness assessment and intervention design are undermined (Bandura, 1977; De La Cruz et al., 2021; Heino et al., 2024; Liu et al., 2018).

A major limitation in current HBM applications is the weak operationalization of cues to action, which are often reduced to passive informational stimuli (e.g., social media exposure, peer influence) and therefore show limited predictive value (Raman et al., 2023). Across the 19 reviewed studies, cues to action were addressed in 14, yet found statistically significant in only seven, underscoring the construct’s inconsistent predictive value across contexts. However, current evidence does not consistently support such extended interpretations. Yet, among university populations, the digital ecosystem—while often functioning as a behavioral scaffold that supports health-related actions—also tends to amplify present bias through increasing reliance on external rewards, such as financial incentives, thereby privileging immediate gratification over long-term behavioral outcomes (Das & Evans, 2014). Empirical evidence shows mixed findings regarding the role of cues to action. Espeño et al. (2024) integrating TPB, HBM, and TEMPA, report that involuntary cues exert little influence on supplement use, while deliberative factors such as attitudes, perceived behavioral control, and health motivation emerge as strong predictors of behavioral intention.

Similarly, studies by Kebede et al. and Raman et al. report limited sensitivity of conventional cue-to-action measures. Kebede’s adaptations from the Look AHEAD Trial relied on attendance metrics and validated physical activity and dietary questionnaires (Kebede et al., 2023), while Raman found that cue-related variables did not significantly predict BMI outcomes (Raman et al., 2023). Multiple adaptations reveal inconsistent balances between internal and external determinants, as well as weight- and gender-stratified responsiveness, underscoring heterogeneous behavioral elasticity and the need for weight-tailored interventions (Saghafi-Asl et al., 2020; Vo & Le, 2025). Some interventions nevertheless show promise. Hou et al. report that a vegetarian meal plan framed as a cue within the HBM predicted measurable changes in body composition (Fishbein, 1979; Hou et al., 2022). In contrast, studies by Lo et al. (2015) and Sheng et al. (2023) indicate that perceived barriers—rather than cues to action—account for a greater share of variance in outcomes observed in metabolic syndrome and student populations, respectively. Repeated adaptation of measurement instruments without adequate cultural recalibration risks compromising both reliability and construct validity, while McArthur’s predefined “healthy” anchors may introduce systematic response bias (Bagozzi & Yi, 1988; Gong & Sheng, 2022; Park, 2011; Wu et al., 2020).

As a result of these findings, cues to action within HBM should be reconceptualized as communicative, context-sensitive, identity-relevant, and economically coherent nudges that engage interpretive frameworks, emotional responses, and normative commitments in order to more effectively catalyze behavioral change (Thaler & Sunstein, 2008). Guan et al. (2025) attribute knowledge–action gaps in Metabolic Dysfunction-Associated Fatty Liver Disease (MAFLD) to low self-efficacy, conflicting health beliefs, weak social support structures, and persistent structural barriers, emphasizing the need for tailored health education and strengthened support systems (Espeño et al., 2024; Guan et al., 2025). Ultimately, public health strategies must shift from technocratic reminders toward institutionally embedded behavioral signaling, where cues communicate shared values and collective purpose. The effectiveness of such cues depends on their timing, framing, and the incentive structures that shape individual agencies. This repositioning aligns with the necessity of fostering socio-institutional trust, where social capital, adaptive capacity, and sociodemographic conditions contribute to systemic behavioral coherence (Fukuyama, 1996; Vâlsan et al., 2025).

The predominance of cross-sectional designs (16 studies) limits the ability to observe longitudinal adherence patterns and causal dynamics. Greater adoption of SEM is therefore warranted because SEM simultaneously estimates latent constructs, accounts for measurement error, and enables the testing of mediation and moderation within integrated pathways—capabilities that first-generation multivariate techniques typically lack (Hair et al., 2021; Carra et al., 2025; Kogevinas & Chatzi, 2021; Solem, 2015; Taris et al., 2021; X. Wang & Cheng, 2020; Sürücü et al., 2023; Xia & Yang, 2019).

In addition, the observed right-skewed and non-normal BMI distributions indicate that population averages obscure important variation across the distribution, particularly at the upper and lower tails where high-risk or treatment-resistant subgroups tend to cluster. This pattern suggests the need for targeted, subgroup-sensitive interventions rather than uniform, population-level approaches (Bogaardt et al., 2024; Huang & Huang, 2023; Lang et al., 2017; Rota et al., 2025; Silverman, 2025).

Empirical findings nonetheless confirm HBM’s structural reliability for predicting weight-management intention while also identifying perceived severity, intention, self-efficacy, cues, barriers, attitude, gender, education, prior dieting and BMI as interrelated predictors. Such relationships are more effectively captured through latent-variable frameworks and longitudinal research designs. Together, these findings suggest that BMI dynamics reflect underlying structural and behavioral asymmetries, requiring adaptive modeling strategies, targeted behavioral interventions, and policy frameworks sensitive to subgroup heterogeneity, rather than continued reliance on cross-sectional, intention-based population averages (Carra et al., 2025; Kogevinas & Chatzi, 2021; Solem, 2015; Taris et al., 2021; X. Wang & Cheng, 2020; Sürücü et al., 2023; Xia & Yang, 2019; Bogaardt et al., 2024; Huang & Huang, 2023; Lang et al., 2017; Rota et al., 2025; Silverman, 2025).

From a complex-systems perspective, behavioral constructs interact nonlinearly and intention-based frameworks, such as TPB and HBM, often fail to capture recursive feedback loops in which behavior reshapes underlying beliefs. Within such dynamics, stress, social pressure, and identity-related cues can function as adaptive triggers, indicating the need for dynamic rather than static behavioral models. Reliance on self-reported measures and cross-sectional study designs further compounds these limitations by reinforcing optimism bias and constraining causal inference. As a result, many studies quantify health beliefs without adequately addressing their strategic implications for intervention design or sustained behavioral change (Carrodano, 2025; Ajzen, 2011; Bales et al., 2023; Bauhoff, 2023; DiMatteo et al., 2025; Taylor et al., 2006).

Composite frameworks that attempt to approximate adaptive systems risk specification bias and ecological fallacy when dynamic processes are represented through static proxies. Such simplifications can obscure nonlinear thresholds, and feedback mechanisms, while also misapplying group-level inferences to individual behavior. When combined with optimism bias, these limitations may generate systemic analytical blind spots, marginalizing cultural, emotional, and identity-based dimensions of behavior. In turn, these distortions can be strategically exploited by commercial actors, who frame health solutions around consumption-oriented interventions while neglecting adaptive, structural, and environmental determinants of behavior (Diez Roux, 2011; Greenland, 2005; Wakefield, 2009; Roy, 2014; Terenzio, 2025; Serttaş et al., 2025; Barankay, 2025; Kukucka & Findley, 2023; Petticrew et al., 2020; Yan et al., 2025; Zhang et al., 2018).

Weight management highlights the structural fragility of behavioral interventions. From a systems-level interpretative perspective, when trust, motivation, or institutional coherence fall below critical thresholds, interventions may collapse rather than gradually decline, while static predictor dashboards remain unable to anticipate such nonlinear dynamics. Addressing this limitation requires resilience-oriented policy architectures capable of tracing feedback loops, identifying critical thresholds, and embedding adaptive signaling alongside identity-sensitive intervention design. This perspective emphasizes that behavioral sustainability is not merely an individual outcome but is deeply embedded in the credibility of health institutions and the strength of social support structures (Fukuyama, 1996; Vâlsan et al., 2025).

From a clinical and practical standpoint, these systemic constraints translate into concrete implications for intervention design and delivery. Weight management programs should move beyond information-based strategies and prioritize the enhancement of self-efficacy as a central intervention target, alongside structured behavioral support mechanisms. In practice, this involves the use of personalized feedback, goal-setting frameworks, and repeated reinforcement strategies, particularly in populations such as university students where behavioral adherence is highly context-dependent. Moreover, effective intervention design must explicitly account for structural barriers, including socioeconomic constraints, time limitations, and environmental access to healthy food options, which often mediate the translation of intention into sustained behavior change. Accordingly, clinical and public health strategies should integrate individual-level behavioral support with broader environmental and policy-level interventions to improve long-term weight management outcomes.

In such contexts, the central challenge shifts from predicting behavior to designing policy frameworks resilient to behavioral variability and systemic uncertainty.

## 6. Conclusions

This study examined the application of HBM in weight management research from 2014 to 2025 using bibliometric and content analyses. The primary objective was to map the intellectual structure of the field and assess why a theoretically mature model often demonstrates limited predictive consistency in applied settings.

The findings showed that, despite the conceptual maturity of the HBM, the field remains geographically fragmented and methodologically heterogeneous. Research is concentrated in a limited number of countries, including China, Iran, and Ethiopia, while relatively weak cross-regional collaboration limits the generalizability of findings. In addition, empirical studies tend to prioritize intention-based outcomes over observed behavioral change, which constrains the model’s explanatory and predictive validity.

A key methodological issue identified in the reviewed studies is the inconsistent operationalization of core HBM constructs, particularly cues to action, which are often weakly defined or indirectly measured. By contrast, self-efficacy emerges as the most consistently supported construct, being statistically significant in 13 of the 17 studies that assessed it. Overall, these findings suggest that the current evidence base primarily captures behavioral intention rather than actual long-term behavioral change.

Empirical evidence indicates that interventions targeting self-efficacy are therefore more likely to be effective, whereas passive informational cues generally underperform unless reconceptualized as identity-relevant, communicative nudges embedded within credible institutional frameworks. From a policy perspective, these findings support strategies that build individual agency, employ context-sensitive cues, and leverage institutional signaling to strengthen trust, while implementing targeted interventions for groups at the extremes of the distribution rather than applying uniform, one-size-fits-all approaches.

The study’s three contributions are supported by the findings. First, the bibliometric analysis clarifies the intellectual structure and thematic evolution of HBM-based weight management research. Second, the content analysis highlights persistent methodological inconsistencies in how HBM constructs are operationalized. Third, the review demonstrates that contextual and institutional factors significantly influence the model’s predictive capacity.

From a methodological perspective, the dominance of cross-sectional designs limits the ability to capture behavioral dynamics over time. Future research should prioritize longitudinal designs and more advanced analytical approaches, including SEM, to better model the complex relationships among constructs.

Several limitations should be acknowledged. One limitation of this review is the inclusion of “structural equation modeling” (SEM) as an inclusion criterion. This decision was made to prioritize studies employing more advanced analytical approaches and providing detailed examinations of relationships among HBM constructs. However, it may have reduced the breadth of the included literature and led to the exclusion of relevant studies that did not use SEM. While this strategy enhanced the analytical depth of the review, it may have reduced its overall comprehensiveness.

A further limitation of this review is that the included corpus exhibits substantial variation in methodological rigor. Although all 19 studies were retained to capture the full breadth of the literature, this heterogeneity may affect the robustness and comparability of findings. Future research should incorporate standardized quality appraisal tools, such as the Mixed Methods Appraisal Tool (MMAT), to better assess study quality and strengthen the validity of synthesized evidence.

Future research should aim to improve the operational consistency of HBM constructs and strengthen the distinction between behavioral intention and actual behavioral outcomes. Practical recommendations include prioritizing the measurement of real behavioral outcomes, adopting standardized measures of self-efficacy and cues to action, and ensuring transparent reporting of SEM analysis. Enhancing measurement standardization while maintaining contextual sensitivity will support the cumulative advancement of HBM-based research in weight management.

## Figures and Tables

**Table 1:** Descriptive Statistics.

Database	No. of Articles	No. of Publishing Sources	Timespan	Document Average Age	Average Citation per Article	Highest No. of Citations per Article
WoS	19	18	2014–2025	3.68	17.11	94

**Table 2:** Co-authorship clusters.

Cluster	Country	Authors’ Research Interest
Cluster 1	Turkey	General and Internal Medicine
Cluster 2	USA	Sport Sciences; Psychology
Cluster 3	Iran	Nutrition & Dietetics; Public, Environmental & Occupational Health
Cluster 4	Ethiopia	Health Care Sciences & Services; Public, Environmental & Occupational Health; Science & Technology
Cluster 5	Egypt	Public, Environmental & Occupational Health; Environmental Sciences & Ecology
Cluster 6	Taiwan	Biochemistry & Molecular Biology; Pharmacology & Pharmacy; Nutrition & Dietetics
Cluster 7	Malaysia	Business & Economics; Social Sciences; Public, Environmental & Occupational Health
Cluster 8	Ethiopia	Science & Technology; Nutrition & Dietetics; Public, Environmental & Occupational Health
Cluster 9	Saudi Arabia	General & Internal Medicine; General & Internal Medicine; Biomedical Social Sciences; Psychiatry
Cluster 10	Iran	Public, Environmental & Occupational Health; General & Internal Medicine; Nursing
Cluster 11	China	Nursing; Cardiovascular System & Cardiology
Cluster 12	Philippines	Food Science & Technology; Computer Science
Cluster 13	Iran	Health Care Sciences & Services

## Data Availability

No new data were created or analyzed in this study. Data sharing is not applicable to this article.

## Abbreviations

The following abbreviations are used in this manuscript:

HBM	Health Belief Model
TPB	Theory of Planned Behavior
BMI	Body Mass Index
WoS	Web of Science
SEM	Structural Equation Modeling
SCT	Social Cognitive Theory
TEMPA	Theory of Effort Minimization in Physical Activity
MMAT	Mixed Methods Appraisal Tool

## Appendix A. Inter-Rater Reliability—Cohen’s Kappa

**Table A1:** Inter-Rater Reliability.

Construct	Observed Agreement (Po)	Cohen’s Kappa (κ)	Interpretation
Perceived Susceptibility	0.842 (16/19)	0.759	Substantial
Perceived Severity	0.842 (16/19)	0.753	Substantial
Perceived Benefits	0.842 (16/19)	0.712	Substantial
Perceived Barriers	0.842 (16/19)	0.712	Substantial
Cues to Action	0.789 (15/19)	0.712	Substantial
Self-efficacy (Diet)	0.842 (16/19)	0.782	Substantial
Self-efficacy (Exercise)	0.842 (16/19)	0.776	Substantial
Overall	0.835 (111/133)	0.753	Substantial

Note: Kappa interpretation follows Landis and Koch (1977): 0.41–0.60 = Moderate; 0.61–0.80 = Substantial; >0.80 = Almost Perfect.

## Appendix B. Systematic Content Analysis and Coding Matrix

**Table A2:** Systematic Content Analysis and Coding Matrix.

Author	Country	Design	Sample (N)	Population	Outcome Type	PSs	PSev	PBen	PBar	CtA	SE-D	SE-E	Behavioral Outcome	Analysis Method	Theory	HBM Total Score (0–21)	HBM Score Reviewer 1 (0–21)	HBM Score Reviewer 2 (0–21)
Espeño et al. (2024)	Philippines	Cross-sectional	250	Fitness users	Consumption	0	0	2	2	0	3	3	Supplement consumption behavior	SEM	HBM + TPB + TEMPA	10	10	9
Kebede et al. (2023)	Ethiopia	Cross-sectional	423	Civil servants	Intention	3	2	3	3	1	2	2	WM intention	CFA + multivariate linear regression	HBM	16	17	16
Raman et al. (2023)	Malaysia	Cross-sectional	440	Adults (Mixed BMI)	Perception + intention	2	3	3	2	3	2	2	WM perceptions & BMI	Regression	HBM	17	17	16
Vo & Le (2025)	Vietnam	Cross-sectional	387	Adults	WM intention	3	3	2	2	2	2	3	WM intention	Logistic regression	HBM	17	17	15
Y. Wang et al. (2022)	China	Cross-sectional	1281	General population	Intention + behavior	3	3	3	3	0	3	0	Healthy eating intention and behavior	PLS-SEM	Extended HBM	15	15	15
Beressa et al. (2025)	Ethiopia	RCT	447	Pregnant women	Behavior	3	2	2	3	2	3	3	Gestational weight gain (GWG)	GSEM	HBM	18	18	16
Abd El Samad et al. (2025)	Egypt	Two-phase (cross-sectional & quasi-experimental)	Phase 1: 800; Phase 2: 100	Medical students	Behavior + intention	3	3	3	3	3	3	3	BMI + Behavioral intention	Quantitative pre-post analysis	HBM	21	21	21
Jorvand et al. (2020)	Iran	Longitudinal	114	Healthcare workers	Behavior	3	3	3	2	1	0	3	Exercise behavior	SEM + RM-ANOVA	HBM	15	15	15
Sheng et al. (2023)	China	Cross-sectional	336	Students	Behavioral intention/Physical activity level	1	3	3	3	3	0	3	Physical Activity (PA)	SEM	HBM	16	16	16
Lo et al. (2015)	China	Cross-sectional	132	At-risk population	Health behavior	2	2	2	3	2	3	3	Health behavior	Hierarchical multiple regression	HBM	17	15	17
Saghafi-Asl et al. (2020)	Iran	Cross-sectional	336	Female students	Intention + Behavior	2	3	3	3	3	3	3	BMI and behavioral intention of weight management (dieting and exercising)	SEM + Hierarchical linear regression	HBM	20	20	20
McArthur et al. (2018)	USA	Cross-sectional	476	Students	Behavior	3	3	3	3	3	0	0	Weight maintenance behavior	One-way ANOVA and regression	HBM	15	15	15
Ahmad et al. (2023)	Malaysia	Cross-sectional	377	Students	Behavioral intention	3	2	3	2	3	3	2	Behavioral intention of weight management	Independent *t*-tests	HBM	18	18	18
Md Nor et al. (2025)	Malaysia	Cross-sectional	404	Married people	Behavioral intention	2	3	2	2	0	0	0	Healthy lifestyle behavioral intention	SEM	HBM + TPB + Body image dissatisfaction + Habit	9	9	9
Wei et al. (2021)	China	Cross-sectional	8840	App users	App adoption	2	2	3	3	0	3	0	App usage behavior (diet, exercise, weight, and login records)	SEM	UTAUT + HBM + Self-control theory + Risk perception	13	12	13
Das and Evans (2014)	USA	Cross-sectional	45	Freshmen students	Perception	1	1	1	1	1	1	1	Students’ perceptions of benefit, barriers and strategies for weight management	Qualitative	HBM	7	7	7
Hou et al. (2022)	Taiwan	Longitudinal	87	Adults	Body composition measurements	3	2	3	3	1	1	1	Changes in body composition (weight, body fat, muscle mass)	GEE + Wald test + regression	HBM	14	14	13
Albasheer et al. (2024)	Saudi Arabia	Cross-sectional	579	Students	Intention	2	2	2	3	3	3	2	Behavioral intention of obesity management	Multivariate logistic regression	HBM	17	16	17
Nategh et al. (2017)	Iran	Cross-sectional	500	Women	Behavior	1	1	3	3	0	0	3	Behavior (Physical activity)	Correlation analysis	Social-ecological model + HBM	11	11	11

PSs = Perceived Susceptibility; PSev = Perceived Severity; PBen = Perceived Benefits; PBar = Perceived Barriers; CtA = Cues to Action; SE-D = Self-efficacy (Diet); SE-E = Self-efficacy (Exercise). Each construct was scored on a 0–3 scale, where: 0—Not mentioned/not measured; 1—Mentioned qualitatively (without statistical analysis); 2—Measured and statistically tested; 3—Statistically significant (reporting an impact on behavior or intention with *p*-value < 0.05). HBM total score ranged from 0 to 21. HBM Total Score represents the final agreed score after discussion. HBM Score Reviewer 1 and HBM Score Reviewer 2 represent the independent scores assigned before consensus was reached.

## Appendix C. Structured Synthesis of Measurement, Reliability, and Outcomes for HBM Constructs Across Included Studies

**Table A3:** Structured Synthesis of Measurement, Reliability, and Outcomes for HBM Constructs Across Included Studies.

Study	HBM Constructs	Measurement Instrument	Reliability (α)	Statistical Method	Outcome Variable	Key Methodological Issue
Espeño et al. (2024)	Perceived benefits, Perceived barriers, Self-efficacy (Diet), Self-efficacy (Exercise)	Questionnaire	0.8–0.9	SEM + reliability analysis	Fitness supplement consumption	Cross-sectional design; Complex integrated model with non-probabilistic sampling; Reliance on self-reported data.
Kebede et al. (2023)	All HBM constructs	Validated HBM questionnaire	0.68–0.89	CFA + multivariable linear regression	Weight management intention	Intention-only outcome; Self-reported BMI bias; Cross-sectional limitation.
Raman et al. (2023)	All HBM constructs	Structured survey	Validated via pilot study (n = 30)	Hierarchical regression + Nonparametric tests (Kruskal–Wallis, Mann–Whitney)	Weight management perception & BMI	Uneven sample distribution; Self-reported height/weight bias.
Vo & Le (2025)	All HBM constructs	Standard questionnaire	0.85–0.94	Multivariable Logistic Regression Analysis	Weight management intention	Convenience sampling; Cross-sectional design; Self-reported data.
Y. Wang et al. (2022)	Susceptibility, Severity, Benefits, Barriers, Self-efficacy, and Health Consciousness	Self-administered questionnaire	0.730–0.897	PLS-SEM	Healthy eating intention and behavior	Cross-sectional design; Self-reported behavior.
Beressa et al. (2025)	HBM	Structured interview-based questionnaire	0.71–0.94	GSEM + SEM	Gestational weight gain (GWG)	Cluster-level randomization; Self-reported dietary intake.
Abd El Samad et al. (2025)	All HBM constructs	Self-administered questionnaire	0.92 (overall)	Paired *t*-test; Chi-square test; Independent *t*-test	BMI + Behavioral intention	Lack of control group; Short-term follow-up (3 months); Convenience sampling.
Jorvand et al. (2020)	Perceived Susceptibility; Perceived Severity; Perceived Benefits; Perceived Barriers; Self-efficacy (Exercise)	HBM-ISCS questionnaire	0.715–0.816 (subscales); 0.746 (overall)	Chi-square, *t*-test; SEM + RAMANOVA + SEM	Exercise behavior	Self-reported data; Lack of dietary control; Influence of psychological moods.
Sheng et al. (2023)	Perceived benefits, perceived subjective barriers, perceived objective barriers, exercise self-efficacy, perceived severity, cues to action	Health Belief Model Scale for Exercise; International Physical Activity Questionnaire-Short Volume (IPAQ-C); Peer Support Scale for Physical Exercise	0.637–0.798 (subscales); 0.762 (overall)	Independent *t*-tests; Pearson correlation analysis; SEM	Physical activity intention	Cross-sectional design; Random sampling at a single university; Self-reported data.
Lo et al. (2015)	Perceived threat (susceptibility + severity), cues to action, perceived benefits, perceived barriers and self-efficacy	Self-administered questionnaire	0.69–0.91	Three-step hierarchical multiple regression	Health-promoting behavior	Cross-sectional design; Recall bias; Convenience sampling.
Saghafi-Asl et al. (2020)	All HBM constructs	Structured questionnaire	0.71–0.92 (subscales); 0.92 (overall)	Chi-square; Kruskal–Wallis; SEM; Hierarchical linear regression	Behavioral intention of weight management	Cross-sectional design; Gender bias; Self-reported data; Selection bias.
McArthur et al. (2018)	Perceived severity, susceptibility, barriers, benefits, and internal and external cues	Online close-ended questionnaire	0.80–0.94	One-way ANOVA, Kruskal–Wallis, Wilcoxon signed rank sum tests, and Generalized Least Squares Regression	Body Mass Index (BMI)	Cross-sectional design; Low response rate (14.4%); Self-reported weight and height data; Overrepresentation of female (71.3%) and white non-Hispanic (86.2%) students.
Ahmad et al. (2023)	All HBM constructs	Questionnaire	0.92 (overall reliability) and 0.93 (pilot study)	Independent *t*-tests	Behavioral intention of weight management (diet therapy and exercise therapy) and BMI	Cross-sectional design; Convenience sampling; Contextual limitations (COVID-19); Response bias.
Md Nor et al. (2025)	Perceived severity, perceived susceptibility, perceived benefits, and perceived barriers.	Integrated scale—Health Belief Model Scale, TPB Scale, Healthy Lifestyle Belief Scale, Body Area Scale and Creature of Habit Scale.	0.84	SEM	Healthy lifestyle behavior	Cross-sectional design; Integrative Model Complexity; Self-reported data.
Wei et al. (2021)	Perceived benefits, perceived barriers, perceived threats (susceptibility and severity) and self-efficacy	Validated app-use scale	0.816–0.95	SEM	App adoption behavior	Single app focus; Cultural specificity; Self-selection bias.
Das & Evans (2014)	All HBM constructs	NGT qualitative method	N/A	Qualitative analysis	Weight perceptions	No quantitative modeling; Self-selection bias;
Hou et al. (2022)	Perceived susceptibility, perceived severity, perceived benefits and perceived barriers.	Dietary behavior scale	>0.7	GEE; Wald test; multiple linear regression and binary logistic regression.	Body composition changes	Limited construct validation; Short duration; Gender imbalance; Study setting.
Albasheer et al. (2024)	All HBM constructs	Questionnaire	0.92 (overall)	Chi-square tests for BMI associations, and Multivariate Logistic Regression	Obesity management intention	Cross-sectional design; Self-reported data; Specific context.
Nategh et al. (2017)	Perceived benefits, perceived barriers, and self-efficacy	Questionnaire	0.69–0.9	Pearson correlation test	Behavior (Physical activity)	Cross-Sectional design; Self-report bias; Specific demographic.
